# Mapping the attractor landscape of Boolean networks with biobalm

**DOI:** 10.1093/bioinformatics/btaf280

**Published:** 2025-05-06

**Authors:** Van-Giang Trinh, Kyu Hyong Park, Samuel Pastva, Jordan C Rozum

**Affiliations:** LIRICA Team, Aix-Marseille University, Marseille 13397, France; Department of Physics, Pennsylvania State University, University Park, PA 16802, United States; Faculty of Informatics, Masaryk University, Brno 60200, Czech Republic; Institute of Science and Technology Austria, Klosterneuburg 3400, Austria; Department of Systems Science and Industrial Engineering, Binghamton University (State University of New York), Binghamton, NY 13850, United States

## Abstract

**Motivation:**

Boolean networks are popular dynamical models of cellular processes in systems biology. Their attractors model phenotypes that arise from the interplay of key regulatory subcircuits. A succession diagram (SD) describes this interplay in a discrete analog of Waddington’s epigenetic attractor landscape that allows for fast identification of attractors and attractor control strategies. Efficient computational tools for studying SDs are essential for the understanding of Boolean attractor landscapes and connecting them to their biological functions.

**Results:**

We present a new approach to SD construction for asynchronously updated Boolean networks, implemented in the biologist’s Boolean attractor landscape mapper, biobalm. We compare biobalm to similar tools and find a substantial performance increase in SD construction, attractor identification, and attractor control. We perform the most comprehensive comparative analysis to date of the SD structure in experimentally-validated Boolean models of cell processes and random ensembles. We find that random models (including critical Kauffman networks) have relatively small SDs, indicating simple decision structures. In contrast, nonrandom models from the literature are enriched in extremely large SDs, indicating an abundance of decision points and suggesting the presence of complex Waddington landscapes in nature.

**Availability and implementation:**

The tool biobalm is available online at https://github.com/jcrozum/biobalm. Further data, scripts for testing, analysis, and figure generation are available online at https://github.com/jcrozum/biobalm-analysis and in the reproducibility artefact at https://doi.org/10.5281/zenodo.13854760.

## 1 Introduction

Biomolecular networks underpin cellular decisions and are essential in genotype to phenotype mapping. They represent the interactions between molecular entities within a cell, such as genes, proteins, and small molecules. Their kinetic parameters, however, are notoriously difficult to measure or estimate. Fortunately, living systems are often qualitatively robust to these parameters (von Dassow *et al.* 2000), motivating widespread use of qualitative modeling in systems biology, with Boolean networks (BNs) being especially popular (Mendoza *et al.* 1999, Albert and Othmer 2003, Gershenson 2004, Wang *et al.* 2012, Rozum *et al.* 2023). First introduced in a gene regulatory context by Kauffman (1969) as a means to study canalization (epigenetic robustness) and the emergence of phenotypic order, BNs consist of interlinked Boolean automata: each automaton’s state (ON or OFF) is dynamically updated by the states of its linked automata according to a fixed update rule. This state evolves (either synchronously or asynchronously) in discrete time steps, eventually converging to one of several attractors (minimal sets of states from which no escape is possible). These attractors then typically correspond to phenotypes of interest. BNs can exhibit ordered, disordered, or critical perturbation responses, which reflects the robustness of their associated biological phenotypes (Derrida and Stauffer 1986, Balleza *et al.* 2008, Park *et al.* 2023). This has important basic science implications, but also biomedical significance: key driver nodes that disrupt undesired phenotypes represent potential drug targets subject to experimental validation (Steinway *et al.* 2014, Meyer *et al.* 2017, Montagud *et al.* 2022, Folkesson *et al.* 2023).

One approach toward understanding phenotype robustness is through the self-sustaining configurations of small sub-networks in a BN, called stable motifs. These correspond to trap spaces within network dynamics—hypercubes in the state-space from which there is no escape (Zañudo and Albert 2013, Klarner *et al.* 2015)—and have analogs in ODEs (Rozum and Albert 2018a, 2018b). A succession diagram (SD), roughly analogous to canalization landscape by Waddington (1942), is a directed acyclic graph that describes how these trap spaces nest within one another, indicating how entering one region of the phenotypic space is predicated on (or forbidden by) entering another (Zañudo and Albert 2013, Rozum *et al.* 2023). The leaf nodes of the SD are the *minimal trap spaces*, each of which contains at least one attractor. Identifying these gives enormous computational advantages and insight into the possible biological behaviors (Klarner *et al.* 2017, Trinh *et al.* 2022a, Rozum *et al.* 2023).

Identifying one BN attractor (resp. all attractors) is NP-hard (resp. #P-hard) because it contains N-SAT as a sub-problem (Mori and Akutsu 2022). Fortunately, biologically significant BNs are often sparse, which can be leveraged in attractor identification algorithms. Still, critical bottlenecks remain that render many biologically important networks intractable. Previously, authors of this work independently explored three approaches to overcoming these bottlenecks.

First, pystablemotifs (Rozum *et al.* 2021b) leveraged parity and time-reversal transformations to extend and accelerate the iterative SD methodology of Zañudo and Albert (2015) and was used to construct the first-ever exact attractor repertoires for genome-scale BNs (Rozum *et al.* 2021a). A limitation of pystablemotifs is its need to frequently compute Blake canonical forms (i.e. all prime implicants) for all update rules and their negations, limiting its use to very sparse networks where such computations are easy. Following pystablemotifs, AEON.py (Beneš *et al.* 2022) was released, using binary decision diagrams along with transition guided reduction (Benes *et al.* 2021) to dramatically improve the efficiency of graph exploration in attractor identification. Finally, and most recently, mts-nfvs was released by Trinh *et al.* (2022a). It uses an alternate scheme, implemented within the trappist library (Trinh *et al.* 2022b), to identify trap spaces via Petri-net encodings, which are easier to compute than the Blake canonical form. Furthermore, it leverages properties of negative feedback vertex sets (NFVS) to more efficiently search for motif-avoidant attractors (Giang and Hiraishi 2021, Trinh *et al.* 2022c). It uses minimal trap spaces and preprocessing heuristics to simplify or avoid reachability analysis in most cases. Still, non-minimal trap spaces and their nesting relationships further improve the method.

Each of these three methods is faster than the last. The algorithm we present here incorporates advantages from each, along with new insights about how to efficiently build SDs, resulting in biobalm. It uses the iterative SD approach of pystablemotifs, efficient rule representation and symbolic state-space searching from AEON.py, and the trap space identification method and NFVS approach of mts-nfvs. We demonstrate substantial speed improvement compared to these prior methods. Our method enables systematic exploration of motif-avoidant attractors in large BNs, exact attractor identification and control in previously intractable experimentally-supported BNs, and analysis of SD scaling in random and nonrandom BNs to provide insight into the emergence of canalization in biology.

## 2 Materials and methods

Here, we first give an overview of key Boolean modeling concepts and notation. More formal details are given in Supplementary Text S1. Then, we give an overview of the methods implemented by biobalm. Details, pseudo-code, and proofs are given in Supplementary Text S2–S5.

### 2.1 Boolean networks

**Figure 1.**
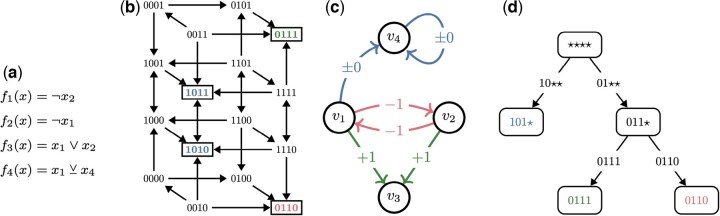
Illustration of the core concepts utilized in this article. (a) A simple ABN $B=\{f_{1},f_{2},f_{3},f_{4}\}$, with ¬ denoting negation, ∧ conjunction, ∨ disjunction, and ⊻ the exclusive or. (b) STG(B). Self-loops are omitted for visual clarity. Attractor states are highlighted and distinguished by color. (c) IG(B). Signs +1 (green), −1 (red), and ±0 (blue) denote positive, negative, and non-monotonic influence, respectively. (d) SD(B). Edges are labeled with the maximal trap spaces that percolate to the target nodes. As is typical (but not guaranteed), the minimal trap spaces (leaf nodes) and attractors (in (b)) coincide.

An asynchronous Boolean network (ABN) of dimension *n*, denoted *B*, is a non-deterministic dynamical system. States of *B* are *n*-dimensional Boolean vectors $x\in B^{n}$, with $x_{v}$ denoting individual vector components. Each network variable *v* is assigned a Boolean update function $f_{v}:B^{n}\to B$ that governs its time evolution. At each discrete time-step, the value of a non-deterministically selected variable is updated to match the output of its update function. When the variables are indexed, we may write $f_{i}$ to refer to $f_{v_{i}}$ and $x_{i}$ to refer to $x_{v_{i}}$. An example ABN with n=4 is shown in Fig. 1a.

The dynamics of ABN *B* are encoded in a state transition graph STG(B) whose nodes are the states $x\in B^{n}$ of *B*. An edge x→y exists in STG(B) if and only if *B* can update from state *x* to state *y* in one time-step (i.e. $f_{v}(x)=y_{v}$ for some *v*). The STG(B) corresponding to the network from Fig. 1a is shown in Fig. 1b. The core feature of each STG(B) is its attractors: minimal subsets of $B^{n}$ that are closed under time evolution. These are also highlighted in Fig. 1b. Note that other BN update schemes exist, such as the synchronous update (see Park *et al.* (2023) for a detailed discussion). However, much of the contributions of this article do not depend on the chosen update scheme (further discussion is given in Supplementary Text S1.2).

ABN dynamics can be viewed as arising from a network of interactions among Boolean automata called an influence graph (IG), denoted IG(B), with nodes $v_{1},\ldots ,v_{n}$. An edge from $v_{i}$ to $v_{j}$ indicates that the state $x_{i}$ of automaton $v_{i}$ is a non-redundant input (In some applications, redundant edges added to IG(B) may be informative, such as those that encode well-known interactions that are nonfunctional within the modeling context. Here, however, we assume that IG(B) only consists of essential interactions and is fully determined by *B.*) to the update function $f_{j}$. The sign of an edge from $v_{i}$ to $v_{j}$ can be −1 for inhibition, +1 for activation, or ±0 if the impact of $v_{i}$ depends on the remaining regulators. Figure 1c shows IG(B) for the example network from Fig. 1a. The IG is very useful for model analysis, as complex dynamics arise from the interplay between positive and negative feedback loops in the IG (Feedback loop sign is determined by the product of its edge signs. Cycles of sign 0 are treated as both positive and negative, because they may act as either, depending on where in the state-space they are evaluated). It is therefore often advantageous to identify a feedback vertex set (FVS), which is a set of nodes that intersects every cycle of IG(B). Controlling the activation of an FVS is sufficient to drive an ABN into any of its attractors (Fiedler *et al.* 2013, Zañudo *et al.* 2017). Furthermore, a NFVS is a set of nodes that intersects every negative feedback loop. Fixing the nodes of any NFVS ensures that all variables in an ABN eventually stabilize (Giang and Hiraishi 2021). Typically, we are interested in an (N)FVS with as few nodes as possible. Identifying the true minimum (N)FVS is computationally difficult, but in our method, a heuristic estimate is sufficient (computed using Beneš *et al.* (2022)). The IG in Fig. 1c has two minimum FVSs: $\left\{v_{1},v_{4}\right\}$ and $\left\{v_{2},v_{4}\right\}$. Meanwhile, the only minimum NFVS is $\left\{v_{4}\right\}$.

### 2.2 Network trap spaces and SDs

Network subspaces represent special subsets of $B^{n}$ given by fixing some of the variables. Formally, subspaces are the members of $B_{*}^{n}$, where $B_{*}=B\cup \{*\}$. The value of a network variable *v* in a subspace $X\in B_{*}^{n}$ can be either fixed ($X_{v}=0$ or $X_{v}=1$) or free ($X_{v}=*$). Each subspace *X* corresponds to a set of states $S(X)\subseteq B^{n}$ that agree with *X* in all fixed variables. For example, X=011∗ corresponds to S(X)={0110,0111}. We refer to *X* and S(X) interchangeably as context allows.

Trap spaces are subspaces that are closed under time evolution. Of special importance are (inclusion) minimal trap spaces, as each is guaranteed to contain at least one attractor. Importantly, however, attractors can also appear outside of the minimal trap spaces. For example, consider a simple ABN $f_{1}(x)=f_{2}(x)=x_{1}⊻x_{2}$. This network has two trap spaces, ∗∗ and 00, of which 00 is minimal. However, the network also has two attractors: $A_{1}=\{00\}$ and $A_{2}=\{01,10,11\}$, meaning that $A_{2}$ does not lie within any minimal trap space. Such attractors are called motif-avoidant (Rozum and Albert 2022). When a motif-avoidant attractor exists, the set of minimal trap spaces is called incomplete (Klarner *et al.* 2015, 2017). Motif-avoidant attractors, as noted by Rozum *et al.* (2021a), Park *et al.* (2023) and Section Benchmarks, are rare, but it is very difficult to rule out their existence a priori. Various methods exist for detecting whether a set of minimal trap spaces is incomplete (Klarner *et al.* 2017, Rozum *et al.* 2021a, Trinh *et al.* 2022c).

The subspace percolation is the process of propagating fixed values among the network variables. We write P(X) to denote the one-step percolation of the subspace *X*. This updates each variable *v* which is free in *X* (i.e. $X_{v}=*$) to a fixed value b∈B if and only if $f_{v}(x)=b$ for every state *x* in *X*. Repeatedly applying the P operator (up to *n* times) results in a subspace where no further variables can be updated, denoted $P^{\infty }(X)$. We say that *X* percolates to the subspace $P^{\infty }(X)$ and that *X* is percolated if $X=P^{\infty }(X)$. For example, consider the ABN from Fig. 1a and the subspace X=∗0∗∗. This one-step percolates to P(X)=10∗∗ because $f_{1}(x)=\neg x_{2}$, which is equivalent to ¬0 for every network state in *X* (since $x_{2}=0$ for every x∈X). Meanwhile, for $f_{3}$ and $f_{4}$, both output values are possible in *X*, hence they remain free. A second application of P yields the subspace 101∗ because $f_{3}(x)=x_{1}\vee x_{2}$ is equivalent to 1∨0 for every state in 10∗∗. Further applications of P result in no additional changes, because $f_{4}(x)$ simplifies to $\neg x_{4}$ in 101∗. Therefore, $P^{\infty }(X)=P^{2}(X)$, i.e. ∗0∗∗ percolates to 101∗.

Knowledge of network’s trap spaces and their relationships with one another can aid in understanding the network’s long-term dynamics, including its attractors (Trinh *et al.* 2022a, Rozum *et al.* 2021b, 2023) and response to interventions (Zañudo and Albert 2013, 2015, Rozum *et al.* 2021a). Percolated trap spaces are typically emphasized because any state in *X* eventually evolves to a state in the percolation of *X*. To formalize relationships between percolated trap spaces, Zañudo and Albert (2013) introduced *SDs*. A SD (We use a novel, simplified definition of SDs. Supplementary Text S1.3.3 gives a detailed comparison to the previous material on this topic.) of an ABN *B*, denoted SD(B), is a rooted, directed acyclic graph. The vertices of SD(B) are exactly all percolated trap spaces of *B*, with the edge relation describing how these nest within one another (by set inclusion). The root node is the percolation of ${*}^{n}$.

Notice that the terminal (leaf) nodes of SD(B) are exactly the minimal trap spaces of *B*. Furthermore, the successors of a node *X* correspond to the trap spaces obtained by percolating trap spaces that are subset-maximal within *X*. Borrowing terminology from related hypergraph structures (Zañudo and Albert 2013, Rozum *et al.* 2021a), we call such maximal trap spaces stable motifs and show them as edge labels of the SD. Most often, every maximal trap space percolates to a distinct SD node. However, in some cases, multiple maximal trap spaces percolate to the same subspace, in which case the edge can be annotated with multiple stable motifs. The SD of the network from Fig. 1a is depicted in Fig. 1d.

### 2.3 Control interventions

SDs are also useful for attractor control, and form the basis for several ABN control algorithms (Zañudo and Albert 2015, Rozum *et al.* 2021b). The majority of these methods involve selecting a path in SD(B) from the root node to a target trap space containing the desired attractor. At each branch point along the path, an intervention is selected to ensure that the system will eventually enter the trap space corresponding to the selected path. The union of these interventions drives the system to the target trap space with probability 1. The advantage of this approach is that it subdivides the attractor control problem into smaller, more manageable pieces. Typically, controlling entry into each trap space along the selected path involves fixing only a small subset of the Boolean variables. In biobalm, we have implemented two control algorithms from Rozum *et al.* (2021b) with only slight modifications to allow for dynamic expansion of the SD (see Supplementary Text S4 for details).

##  

### 2.4 SD construction

We introduce several innovations to the algorithms of Zañudo and Albert (2013) and Rozum *et al.* (2021a, 2021b). At a high level, biobalm is broadly similar to the previous tools (Supplementary Text S2.3): the root node $P^{\infty }({*}^{n})$ of the ABN *B* is established by percolating the trivial trap space ${*}^{n}$, and it is stored in a digraph SD(B). Then, SD(B) is further expanded by selecting a node (percolated trap space) *X* in SD(B), identifying all maximal trap spaces within *X*, and percolating them to obtain the successor nodes. These are incorporated into SD(B). Once the maximal trap spaces are computed for all nodes, the digraph SD(B) is the SD of *B*, as introduced in Section 2.

Compared to previous tools, however, biobalm has several key advantages. First, we use a more efficient trap space identification method by Trinh *et al.* (2022c, a), which we further improved by implementing a heuristic for achieving more compact encodings of update functions (Supplementary Text S2.1). Second, we have implemented a more efficient percolation function that avoids the expensive step of recomputing prime implicants (Supplementary Text S2.2). Third, we adapt the attractor identification method of Trinh *et al.* (2022a) to apply to arbitrary percolated trap spaces instead of only minimal trap spaces (Supplementary Text S3.3). Finally, we decouple attractor identification from SD construction and implement schemes for partially expanding the SD (discussed further in Section Attractor identification).

### 2.5 Partial expansion strategy

Previous methods (Zañudo and Albert 2013, Rozum *et al.* 2021b) construct SD(B) by preferentially expanding deeper nodes. In biobalm, we implement multiple strategies, including depth-first and breadth-first expansion, allowing early termination if certain size or depth threshold is exceeded. We also developed several partial expansion strategies, which produce a sub-graph of SD(B) in which certain nodes are not expanded, meaning their child nodes in SD(B) are omitted. Upon completion, these methods produce partial SDs that are functionally equivalent to the full SD for attractor identification and control, but which eliminate computationally expensive (and cognitively burdensome) redundancies. Of these strategies, the one that removes the most redundancies, and which we have selected as the default method for biobalm, is the source block expansion (Supplementary Text S5). Conceptually, this method is similar to Su and Pang (2021) or Kadelka *et al.* (2023) in that it identifies hierarchies of sub-networks (blocks of variables) that are mutually independent and can be processed separately, thereby eliminating redundancy associated with permuting the order of trap space entry. Full details are given in Supplementary Text S5.

**Figure 2.**
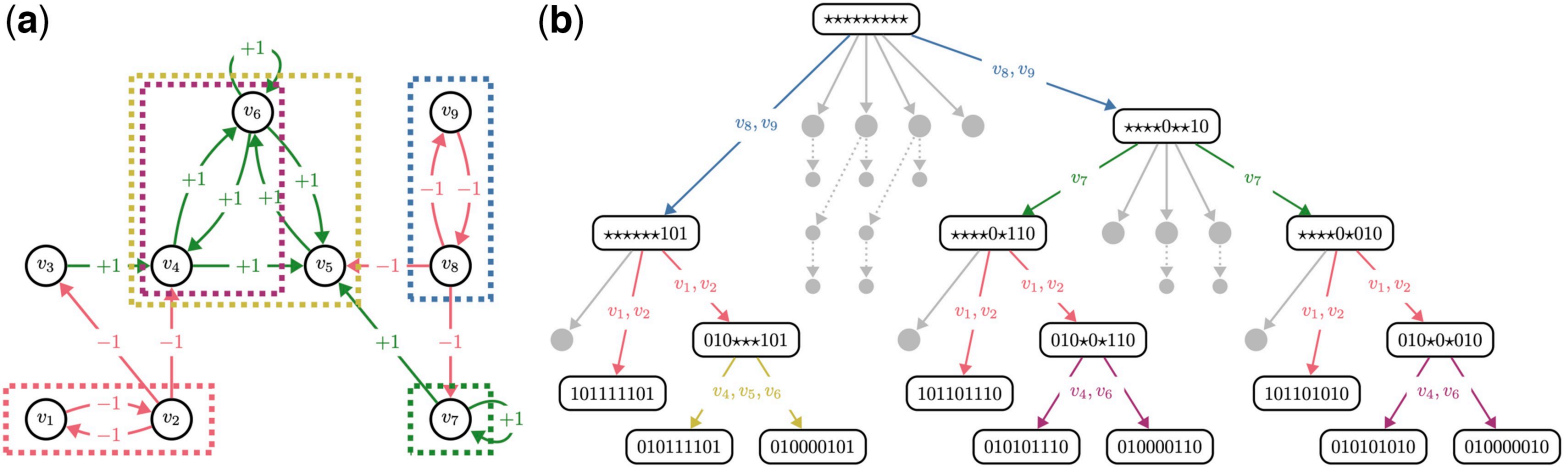
Example of a partial SD expansion by independent source blocks: (a) The IG of the simplified BN from Sánchez and Chaouiya (2016) (see Supplementary Text S8.2 for details), with relevant variable blocks highlighted; (b) The partially expanded succession diagram. Colored edges are labeled with the expanded source blocks. Larger grey nodes represent trap spaces that are discovered but never expanded. Smaller grey nodes are never discovered, but appear in the full SD. For brevity, only one edge is shown per each non-expanded node.

In Fig. 2, we show the application of this method to the mammal sex-determination network developed in Sánchez and Chaouiya (2016) with input values set to true (see also Supplementary Text S8.2). In the root node of the SD, there are six maximal trap spaces divided among four source blocks: $C_{1}=\{v_{8},v_{9}\}$, $C_{2}=\{v_{1},v_{2}\}$, $C_{3}=\{v_{7},v_{8},v_{9}\}$, and $C_{4}=\{v_{1},\ldots ,v_{9}\}$. Initially, we choose $C_{1}$ (blue), which in one branch also eliminates $C_{3}$. In the other branch, we then pick $C_{3}$ (green), which is now reduced to just $v_{7}$ ($v_{8}$ and $v_{9}$ are already fixed by $C_{1}$). Next, we expand $C_{2}$ (red). This either leads directly into a minimal trap space, or it simplifies $C_{4}$ to just two (purple) or three (yellow) variables, depending on the choice in $C_{1}$. Notice that we expanded much fewer SD nodes (17) compared to the full SD (36). Also, the largest network ever considered in the attractor identification has only three variables instead of nine.

### 2.6 Attractor identification

In biobalm, we consider two variants of the attractor identification problem: First, the attractor sets problem is to determine every set of states that represents an attractor of network *B*. This is how the attractor identification is typically understood, but it means that each attractor set must be sufficiently small such that it is fully identifiable. As this is not always the case for large complex attractors, we also consider the attractor seed problem, which is to identify exactly one representative seed state for each attractor. This allows us, e.g. to identify the presence of motif-avoidant attractors without fully enumerating their states.

**Figure 3.**
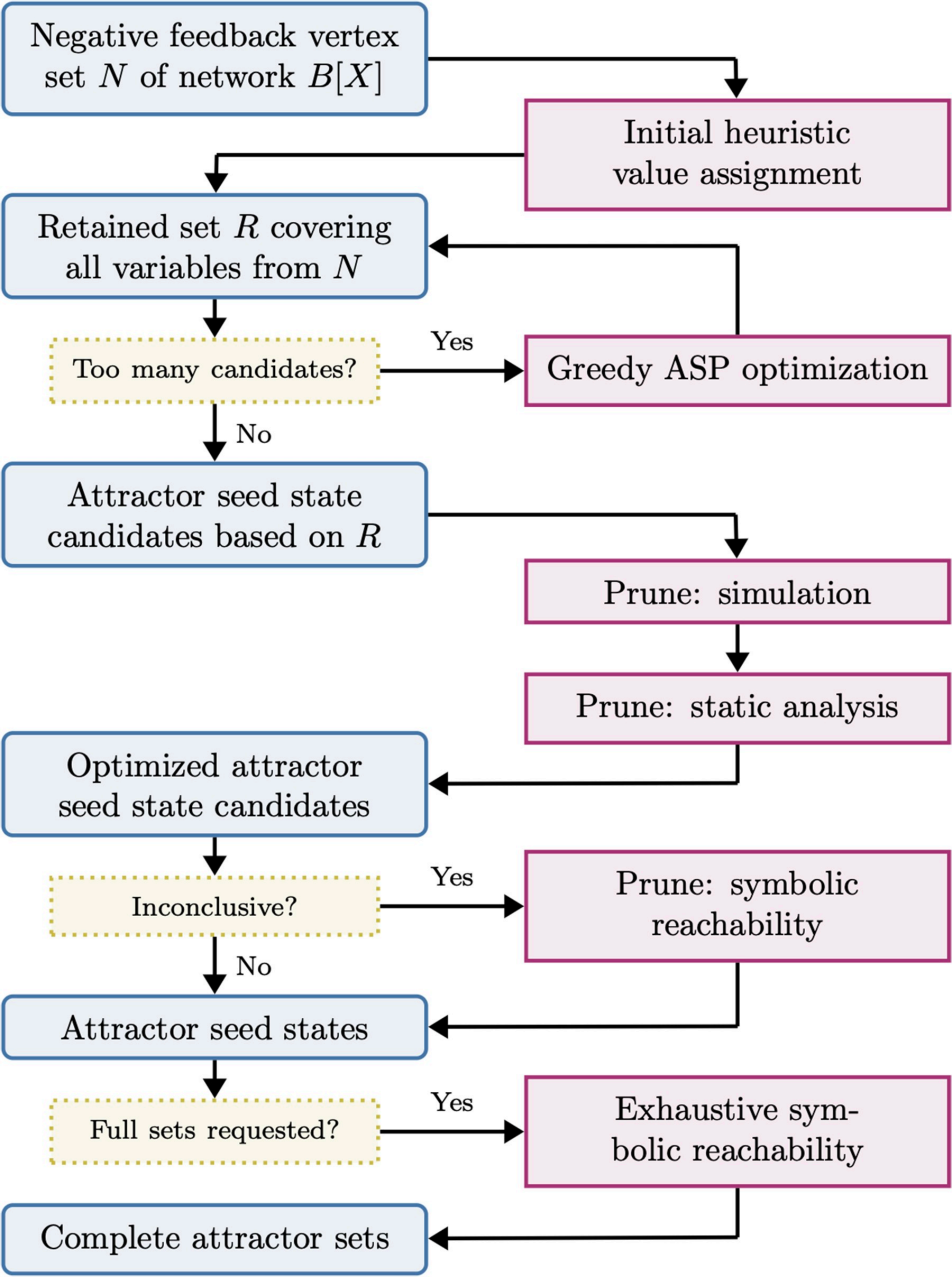
Attractor identification workflow for a fixed node (trap space) *X* of the succession diagram SD(B). Blue (squared) and purple (rounded) boxes represent data and computational steps, respectively.

Our approach in biobalm is based on the method of mts-nfvs by Trinh *et al.* (2022a), which we have significantly improved and extended to apply to arbitrary percolated trap spaces rather than only the minimal ones. Our workflow applies to each SD node *X* individually. A summary is given in Fig. 3 as well as Supplementary Text S3.

Recall that attractors cannot cross trap space boundaries, and that the T(A) (smallest trap space that contains *A*) for each attractor *A* is always a fully percolated trap space. Thus it is sufficient to search for attractors only within percolated trap spaces, i.e. the nodes of SD(B). Furthermore, when searching an SD node *X* for attractors, we can disregard not only the states outside *X*, but also the states in each successor node *Y* (as these are considered separately when searching *Y*). We use the NFVS-based method of Trinh *et al.* (2022a) to identify candidate attractor seeds in each percolated trap space, and then verify or eliminate them using randomized simulation, static analysis (using pint; Paulevé 2017), or symbolic reachability (using AEON.py; Beneš *et al.* 2022).

## 3 Results

### 3.1 Benchmarks

**Figure 4.**
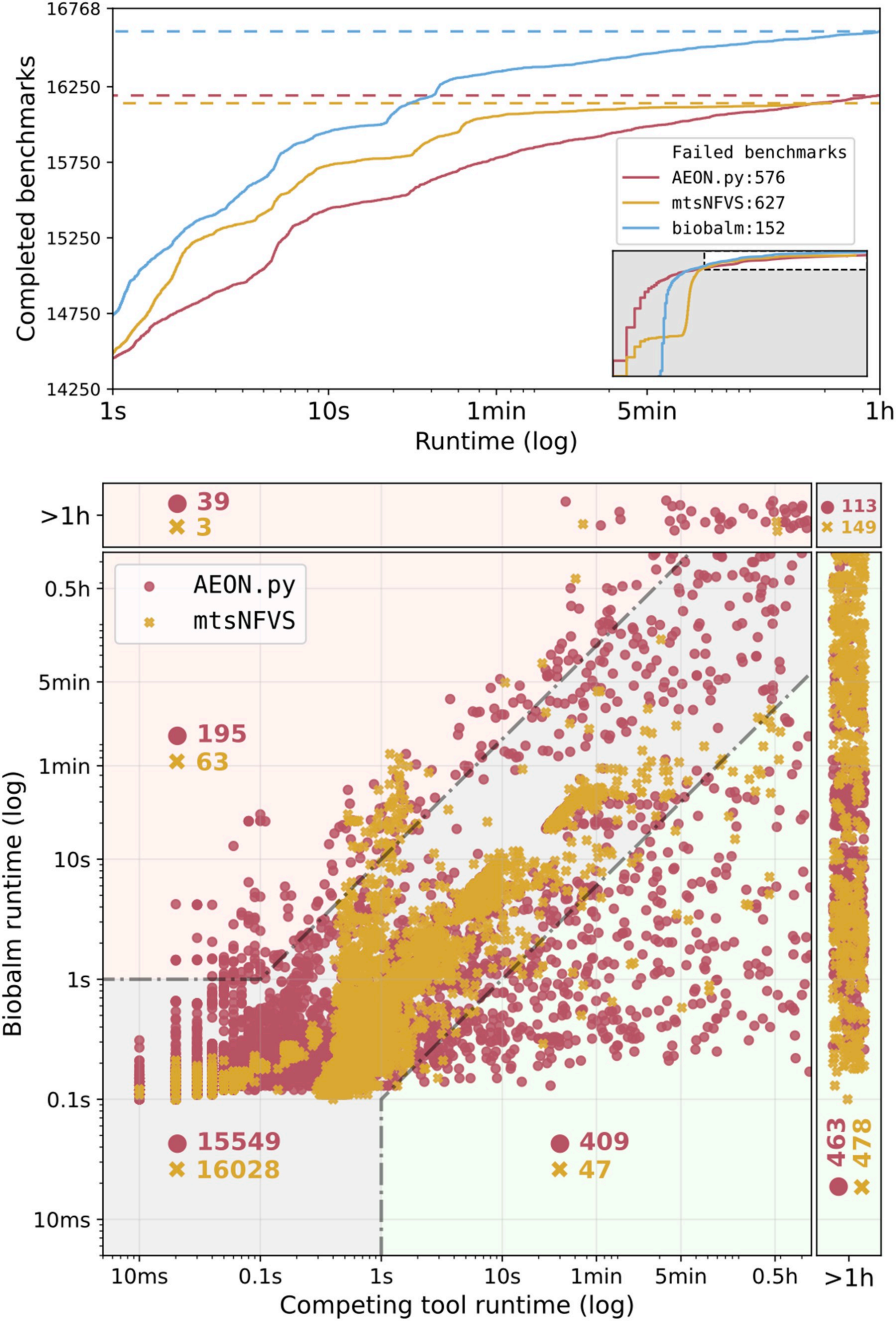
Performance comparison of biobalm versus AEON.py and mts-nfvs for attractor identification. (Top) The total number of completed benchmarks within 1 h timeout (vertical axis) out of our test ensemble of 16 768 benchmark models as a function of time (horizontal axis, logarithmic scale). We show times >1 s in the main panel; the full range is shown in the inset and in Supplementary Text S6. (Bottom) Runtime of individual benchmark instances. Timeouts are placed at the margins of the plot, indicated by the “ >1 h” labels. Green (red) regions represent results where biobalm was at least 10× faster (slower) than the competing tool and the slower tool took longer than 1s to complete. The grey region contains instances where tools performed similarly. The number of cases that fall into each region is indicated in red or yellow text for AEON.py and mts-nfvs, respectively.

To evaluate the overall effectiveness of biobalm, we consider the attractor seed identification problem over a large collection of real-world (230 networks with 14 010 input configurations; from the BBM dataset; Pastva *et al.* 2023) and synthetic (2760 networks; critical N-K, nested canalyzing, and dense ensembles) Boolean networks. A detailed description of experiment setup is given in Supplementary Text S6. We compare biobalm with AEON.py (Beneš *et al.* 2022) and mts-nfvs (Trinh *et al.* 2022a). Figure 4 summarizes the attractor benchmark results. The top panel shows the number of benchmarks completed as a function of time. The bottom panel compares the performance on individual benchmarks. In Supplementary Text S6, we also provide an extended versions of Fig. 4, stratified across individual tools and network ensembles. Importantly, we have not encountered any motif-avoidant attractor in either of ensembles.

We have not included pystablemotifs in the attractor identification benchmarks because Beneš *et al.* (2022) demonstrate that AEON.py is superior for this task. However, we have tested pystablemotifs against biobalm on a smaller set of real-world models to evaluate SD expansion and control. Results are provided in Supplementary Figs S9 and S10. In these tests, biobalm completed almost all benchmarks at least 10× faster than pystablemotifs.

### 3.2 Attractor landscape ensembles

To demonstrate the utility of biobalm, we have compared the full SD structure of 230 ABN models of cell processes from the BBM dataset (Pastva *et al.* 2023), in 14 010 parameter configurations, to the SDs of 69 000 random ABNs drawn from three null model ensembles. The null model ensembles include two ensembles of critical N-K models (Kauffman 1969) with in-degree *K* = 2 and *K* = 3, and an ensemble of nested canalizing function (NCF) networks generated using the methods of Newby *et al.* (2022). The NCF networks have nested canalizing regulatory functions—meaning inputs determine outputs in a hierarchical manner—and topology matched to biological networks as reported by Kadelka *et al.* (2024). We generated 100 random networks with equal size matched to each BBM model, thereby resulting in three ensembles of 23 000 networks each.

**Figure 5.**
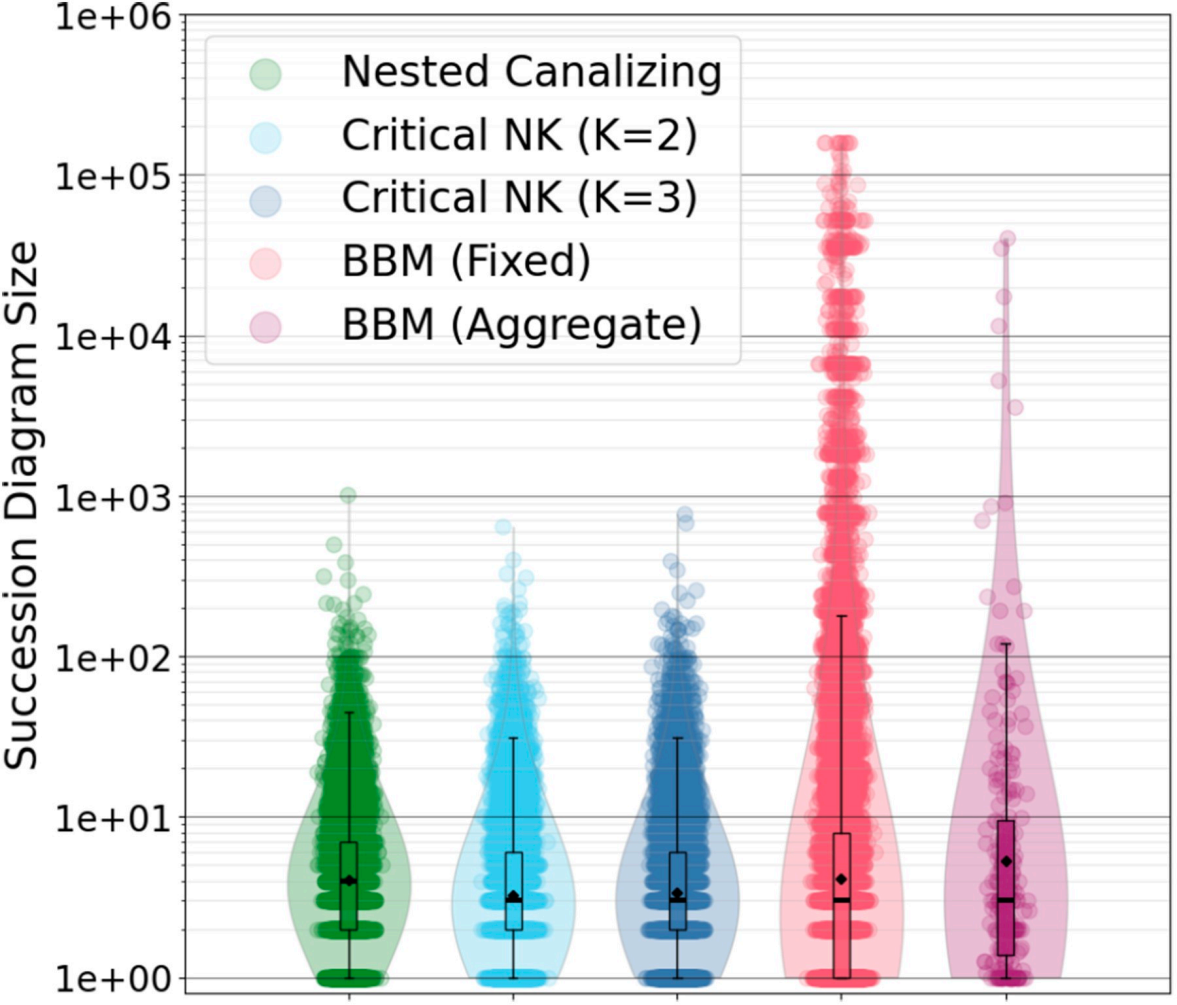
Distributions of succession diagram size (number of nodes in the fully-expanded succession diagram) for various ABN ensembles. BBM networks with random input configurations (up to 128 independent samples) are shown in red, with the average succession diagram size across these samples for each network depicted in purple. Random networks of different types are indicated in green, cyan, and dark blue, and are constructed to match the distribution of the number of variables in the BBM ensemble. Gaussian noise is added to the horizontal position of each point. Box plots and density plots are computed in log space.

As shown in Fig. 5, the distribution of SD sizes for empirical models is more heterogeneous and has a longer tail than for our three ensembles of random networks. The mean, variance, and kurtosis of the BBM distribution are statistically significantly higher than for random networks (via bootstrapped 95% confidence intervals—see Supplementary Text S7.3). We observe a similar pattern in the number of attractors (see Supplementary Table S2). Additionally, we controlled for any automatically-generated models within BBM and observed similar results regardless of the model origin (Supplementary Text S7.4).

We observe that SD size and the number of attractors grow exponentially with SD depth (denoted *d*) in all four ensembles (Supplementary Fig. S13). Note that the SD is free to have any depth, any size, and any number of attractors. SD size is roughly bounded by $3^{d}$, and the number of attractors is roughly bounded by $2^{d}$, scaling bounds that correspond to networks of independent bistable cycles. Moreover, SD size as a function of depth is approximately bounded between the scaling obtained for uniform trees with three children and one child (linear chains), with many SDs falling near the scaling law obtained for uniform binary trees (two decisions for each SD node). These observations suggest that attractor landscapes are typically not broad and shallow—instead, attractor commitment arises from a series of decisions between a small number of possible choices.

## 4 Discussion

Extracting biological insights from a Boolean network requires a strong understanding of its range of possible behaviors, as well as of the circumstances under which they arise. Despite recent major advances, the problem of identifying all the attractors in a large, dense ABN remains difficult. This problem is increasingly important as modelers of cell processes develop ever larger and more complete networks (and integrate them into population-level models). We have previously contributed to this problem individually (Rozum *et al.* 2021b, Beneš *et al.* 2022, Trinh *et al.* 2022a), and here, we present a combined and significantly improved approach in the Python 3 library biobalm, the biologist’s Boolean attractor landscape mapper.

The attractor identification benchmarks we have presented demonstrate a substantial improvement over AEON.py (Beneš *et al.* 2022) (which has been previously demonstrated to significantly outperform pystablemotifs by Rozum *et al.* (2021b)) and a moderate speed improvement over mts-nfvs (Trinh *et al.* 2022a). Crucially, biobalm successfully analysed 463 networks and 478 networks where AEON.py and mts-nfvs failed, respectively. Out of these, 75 networks were uniquely solved by biobalm (27 biological and 48 random). With a more generous timeout of two days, biobalm only fails 26 benchmarks, primarily in large models with more than 100 000 complex attractors that are limited by the time needed to enumerate individual attractors. Overall, biobalm is the fastest and most robust among the tested tools. Moreover, biobalm is simultaneously computing the SD, and therefore yields a much more informative output that describes the decision points in the network circuitry and can be used to perform computationally efficient attractor control using the control algorithms introduced in Rozum *et al.* (2021b). Compared to pystablemotifs, this results in a significant (10× or better) speed-up in control strategy identification.

Importantly, biobalm provides a modular approach to both SD construction and attractor identification, enabling multiple methods and heuristics informed by the SD (Fig. 3). This allows to easily replace components (NFVS computation, minimal trap space computation, symbolic reachability, etc) or introduce new methods as improvements become available.

In addition to benchmarks, we have used biobalm to study the distribution of SD sizes in biological models from the Biodivine Boolean Models (BBM) repository (Pastva *et al.* 2023), the largest curated collection of biological Boolean networks currently available. We compared these models to similarly sized critical Kauffman networks (Kauffman 1969) (*K* = 2 and *K* = 3) as well as to an ensemble of canalizing random networks generated using the method of Newby *et al.* (2023). We observe that SD depth scaling is consistent with attractor landscapes constructed from a series of small decisions, lending support to the hypothesis that biomolecular networks exhibit modular, hierarchal structure (Sales-Pardo *et al.* 2007, Park *et al.* 2023). We also find that the distribution of SD size for published Boolean network models with fixed inputs is highly heterogeneous and contains extremely large SDs (several thousand nodes), as compared to the SD distributions for three biologically-inspired random model ensembles. Allowing inputs to vary in the published models would even further accentuate these differences. This suggests that, even accounting for network size, degree distribution, and the prevalence of canalizing functions in biological Boolean networks (Kadelka *et al.* 2024), they exhibit more complicated Waddington canalization landscapes at the system-level. Because the trap spaces that compose the SD arise from positive feedback loops (Zañudo and Albert 2013), we propose that non-local topological features of the IG (such as prevalence and overlap of cycles) play a significant role in the emergence of complex Waddington landscapes. It seems likely that local features (such as various measures of canalization in individual update functions; Correia *et al.* 2018) are not sufficient to explain or predict the complexity of the attractor landscape—though they may still be informative in computing non-local metrics, e.g. via the effective graph (Gates *et al.* 2021). Testing our hypothesis requires a deeper analysis of this phenomenon and remains as future work.

## Supplementary Material

btaf280_Supplementary_Data

## Data Availability

The tool biobalm is available online at https://github.com/jcrozum/biobalm. Further data, scripts for testing, analysis and figure generation are available online at https://github.com/jcrozum/biobalm-analysis and in the reproducibility artefact at https://doi.org/10.5281/zenodo.13854760.
